# Hope As A Motivator For Healthy Behaviors In Older Adults: Findings From A Cross-Sectional Survey

**DOI:** 10.1093/geroni/igab046.3611

**Published:** 2021-12-17

**Authors:** Mushira Khan, Patrick Hill, Catherine O'Brien, Dugan O'Connor

**Affiliations:** 1 Mather Institute, Plainfield, Illinois, United States; 2 Washington University in St. Louis, St. Louis, Missouri, United States; 3 Mather, Evanston, Illinois, United States

## Abstract

Hope can be understood as a motivational state that enables people to move toward their goals. Yet, how hope may act as a motivator for healthy behaviors in older adults is not well-understood. Further, the extant literature utilizes varied conceptualizations of hope, and a better understanding of the constructs that underlie the relationship between hope and health behaviors is needed. This study examined the relationship between hope and health behaviors, explored how this relationship may differ across different socio-demographic groups, and considered how hope relates to perceived future selves among older adults. Community-dwelling adults 55 years and older (n = 711; mean age 67.38 years; 280 men, 431 women) completed an online, cross-sectional survey. Survey measures included, along with the Adult Hope Scale (AHS) and the Herth Hope Index (HHI), a health behaviors checklist, self-reported health, and a future self scale. We found a moderately strong positive correlation between hope and healthy behaviors in older adults (AHS r = 0.46, p < .01; HHI r = 0.50, p < .01). Participants with higher levels of hope also reported more positive future selves and better health. The associations were similar across different racial/ethnic groups and the magnitude of this effect held even after controlling for gender, education, marital status, and income. Of the two hope scales, we recommend the AHS measure given its relative parsimony, greater use in the field, and the fact that the associations were fairly similar to the HHI with respect to health and health behavior.

